# NEK2 mediates ALDH1A1-dependent drug resistance in multiple myeloma

**DOI:** 10.18632/oncotarget.2388

**Published:** 2014-12-09

**Authors:** Ye Yang, Wen Zhou, Jiliang Xia, Zhimin Gu, Erik Wendlandt, Xin Zhan, Siegfried Janz, Guido Tricot, Fenghuang Zhan

**Affiliations:** ^1^ Departments of Internal Medicine, University of Iowa Carver College of Medicine, Iowa City, United States; ^2^ Vanderbilt University, Nashville, Tennessee, United States; ^3^ Departments of Pathology, University of Iowa Carver College of Medicine, Iowa City, United States

**Keywords:** Plasma cell myeloma, aldehyde dehydrogenase, NIMA-related kinase, tumor-initiating cell

## Abstract

We reported previously that increased expression of aldehyde dehydrogenase 1 (ALDH1) in multiple myeloma (MM) is a marker of tumor-initiating cells (TICs) that is further associated with chromosomal instability (CIN). Here we demonstrate that member A1 of the ALDH1 family of proteins, ALDH1A1, is most abundantly expressed in myeloma. Enforced expression of ALDH1A1 in myeloma cells led to increased clonogenicity, tumor formation in mice, and resistance to myeloma drugs *in vitro* and *in vivo*. The mechanism underlying these phenotypes included the ALDH1A1-dependent activation of drug-efflux pump, ABCB1, and survival proteins, AKT and BCL2. Over expression of ALDH1A1 in myeloma cells led to increased mRNA and protein levels of NIMA-related kinase 2 (NEK2), whereas shRNA-mediated knock down of NEK2 decreased drug efflux pump activity and drug resistance. The activation of NEK2 in myeloma cells relied on the ALDH1A1-dependent generation of the retinoid X receptor α (RXRα) ligand, 9-cis retinoic acid (9CRA) – not the retinoic acid receptor α (RARα) ligand, all-trans retinoic acid (ATRA). These findings implicate the ALDH1A1-RXRα-NEK2 pathway in drug resistance and disease relapse in myeloma and suggest that specific inhibitors of ALDH1A1 are worthy of consideration for clinical development of new approaches to overcome drug resistance in myeloma.

## INTRODUCTION

Multiple myeloma (MM), a difficult-to-treat and in most cases incurable neoplasm of the hematopoietic bone marrow, is characterized by clonal expansion of malignant, antibody-producing plasma cells. MM is the second most common blood cancer, accounts for ~1–2% of newly diagnosed cancers overall, and is disproportionately represented in the elderly population [1]. Myeloma cells exhibit numerous gene expression changes and cytogenetic aberrations that frequently affect the immunoglobin heavy-chain locus on chromosome 14 as well as loci on chromosomes 1, 13 and 17 [2–5]. Abnormalities of this sort underlie not only aggressive disease resulting in poor clinical outcome, but also promote acquisition of drug resistance by myeloma cells. With regard to the mechanism of drug resistance in myeloma, Greenman *et al.* implicated deregulated protein kinases [6]. Additional drivers of drug resistance include aberrant expression of transcription factors, mutations in tumor suppressor genes and distorted cell cycle regulation [7]. Despite these advances, additional research is warranted to enhance our understanding of the genetic pathways of myeloma drug resistance.

The recent discovery of drug-resistant tumor subclones in patients with myeloma [8–10] has shed light on the long-standing clinical observation that the response to myeloma chemotherapy is often heterogeneous and sometimes even lesion-specific [11]. Overcoming drug-resistant myeloma in the clinic is a serious challenge that requires new approaches based on results from high-throughput proteomic and genetic analysis tools, such as global gene expression profiling (GEP), RNA sequencing and whole-exome or whole-genome sequencing, which must be combined with sophisticated bioinformatics and biostatistics algorithms [12]. Our group has recently performed sequential GEP analysis of myeloma samples at baseline (newly diagnosed disease), in the course of high-dose chemotherapy and tandem autologous stem cell transplantation (ASCT), and at relapse. This effort uncovered 56 genes tightly associated with drug resistance and rapid disease relapse in myeloma. Intriguingly, 10 of the top 20 genes fell into a well-established chromosomal instability (CIN) signature of cancer [13]. Additionally, we recently reported that ALDH1, a marker of myeloma initiating cells (TICs), is also linked to the CIN signature [14]. High expression of this signature has been shown to predict poor clinical outcome and confer multidrug resistance (MDR) to myeloma and other forms of cancer [13, 15, 16]. The mechanism linking ALDH1 and CIN with MDR has not yet been established.

The human genome contains 19 aldehyde dehydrogenase-encoding ALDH genes and 3 pseudogenes [17]. Aldehyde dehydrogenases are not only crucial for protecting cells from toxic aldehydes, but are also known to play important roles in cancer development, retinoic acid metabolism and drug resistance [17]. For example, high activity of ALDH1A1 and ALDH2 increases the risk of ethanol-induced cancers [18, 19]. ALDH2 is required for embryo survival and early morphogenesis in mice [20]. In humans, deletions of ALDH3A1 or ALDH3A2 cause Sjogren-Larson syndrome [21]; mutations in ALDH4A1 underlie type II hyperprolinemia [22]; mutations in ALDH5A14 cause mental retardation, ataxia and seizures [23]; and allelic variants of ALDH18A1 result in hyperammonemia [24]. Because of their importance for drug metabolism and oncogenesis, ALDH1, ALDH2 and ALDH3 are the most extensively studied members of the ALDH family of enzymes [25, 26]. Normal cells contain two ALDH1 isoforms, ALDH1A1 and ALDH3A1, but the expression of these proteins has also been associated with drug resistance in cancer stem cells (CSCs) [27]. The Aldefluor assay, which permits investigators to detect and separate ALDH1-expressing cells from cells that lack ALDH1 expression, has led to a wave of cancer studies that have implicated ALDH1 in drug resistance of adenocarcinoma of lung [28], melanoma [29], breast cancer [30] and hematopoietic tumors, such as myeloma [8, 10].

This study took advantage of the Aldefluor assay to elucidate the role of ALDH1 in MDR in myeloma in greater depth. We show that A1 is the dominant isoform of ALDH1 in MM. Enforced expression of ALDH1A1 in myeloma cells led to increased activity of the drug efflux pump, ABCB1, and to more vigorous tumor growth in mice. We also demonstrate that over-expression of ALDH1A1 in myeloma leads to elevated NEK2 levels, using a mechanism that includes 9-cis retinoic acid-dependent RXRα signaling [31]. Taken together, our results support the notion that ALDH1A1 is a promoter of drug resistance in myeloma that is worthy of consideration for therapeutic targeting in order to overcome MDR and improve the outcome of patients with myeloma.

## RESULTS

### Increased expression of *ALDH1A1* in serial myeloma samples from the same patients

**Figure 1:**
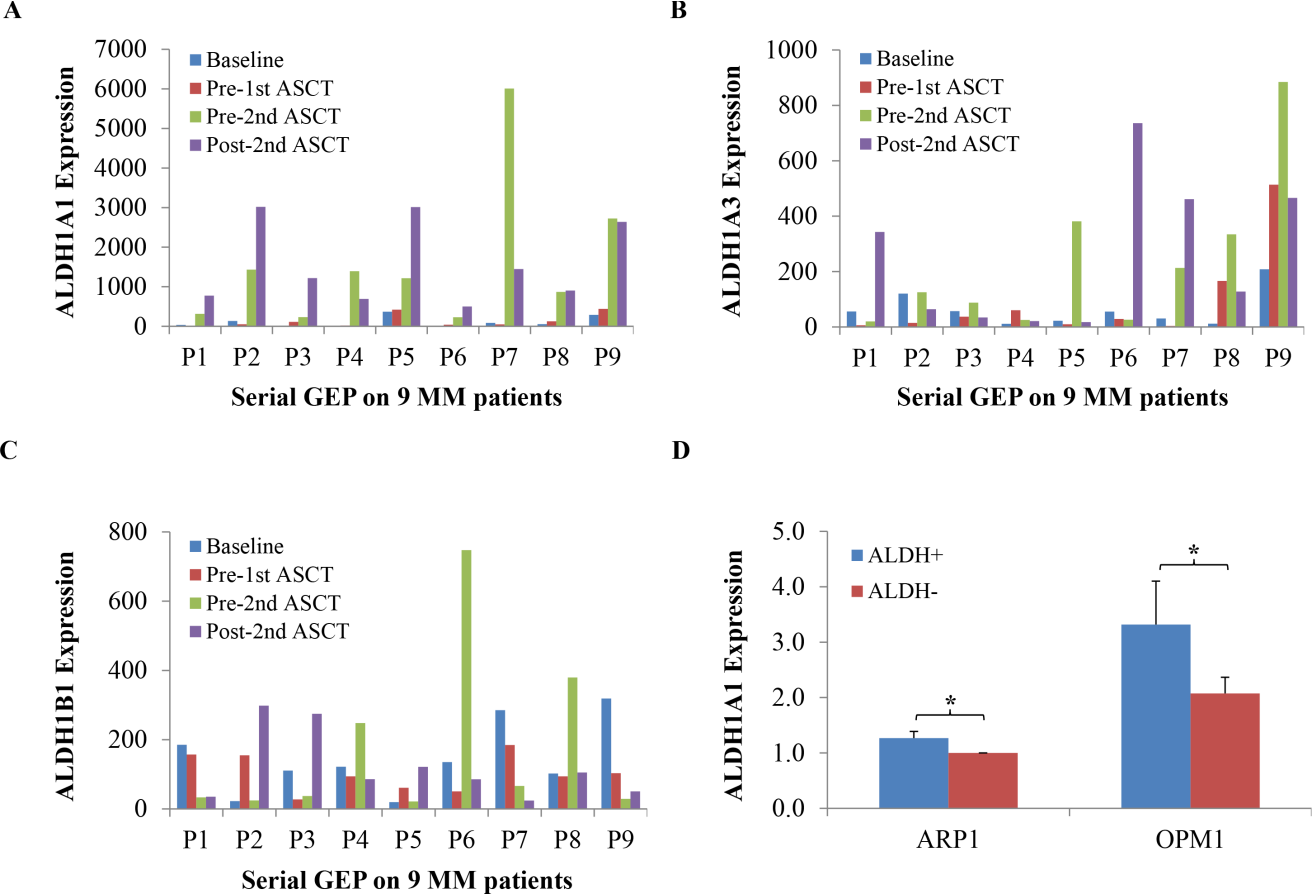
Up-regulation of *ALDH1A1* in the course of myeloma therapy and progression **(A-C)** Message levels of *ALDH1A1, ALDH1A3 and ALDH1B1* in myeloma patient samples collected at diagnosis (blue bars), pre-1^st^ (red), pre-2^nd^ (green) and post-2^nd^ (purple) autologous stem cell transplant (ASCT) were determined by Affymetrix U133 Plus2 microarray analysis and plotted. A total of 9 patients, designated P1-P9, were investigated. **(D)** Levels of mRNA of *ALDH1A1* are plotted. Gene expression was measured using qPCR (*, p<0.05).

To evaluate whether the expression of *ALDH1* exhibits changes in the course of myeloma therapy and disease progression, we queried the mRNA levels of the three most well-studied members of the *ALDH1* gene family, *ALDH1A1*, *ALDH1B1*, and *ALDH1A3*, in 9 patients with myeloma for which the following four serial, microarray-based gene expression profiles (GEPs) were available: at diagnosis, prior to the first and second autologous stem cell transplant, and after the second transplant. Message levels of *ALDH1A1*, but not of the other family members, increased substantially in 9 of 9 (100%) patients (Figure 1A-C). This result not only indicated that *ALDH1A1* is the predominant form of ALDH1 in myeloma, but also suggested that the gene is upregulated in response to standard-of-care myeloma therapy. Next, we sought to determine whether *ALDH1A1* is also the chief representative of the *ALDH1* family in two human myeloma cell lines (HMCLs) that were selected for mechanistic studies on myeloma drug resistance: ARP1 and OPM1. We took advantage of the Aldefluor® assay, which relies on flow sorting to fractionate cells that exhibit ALDH1 activity (designated ALDH1^+^) from cells that do not (ALDH1^−^) [14], to separate ARP1 and OPM1 cells according to ALDH1 status and then used qPCR analysis to show that *ALDH1A1* is more highly expressed in ALDH1^+^ than ALDH1^−^ cells (Figure 1D). Differences of this sort were not observed for *ALDH1B1 and ALDH1A3* (not shown). This finding encouraged us to use ARP1 and OPM1 cells as experimental model system to evaluate the mechanisms by which ALDH1A1 promotes drug resistance in myeloma.

### Over-expression of ALDH1A1 promotes resistance to myeloma drugs *in vitro*

**Figure 2:**
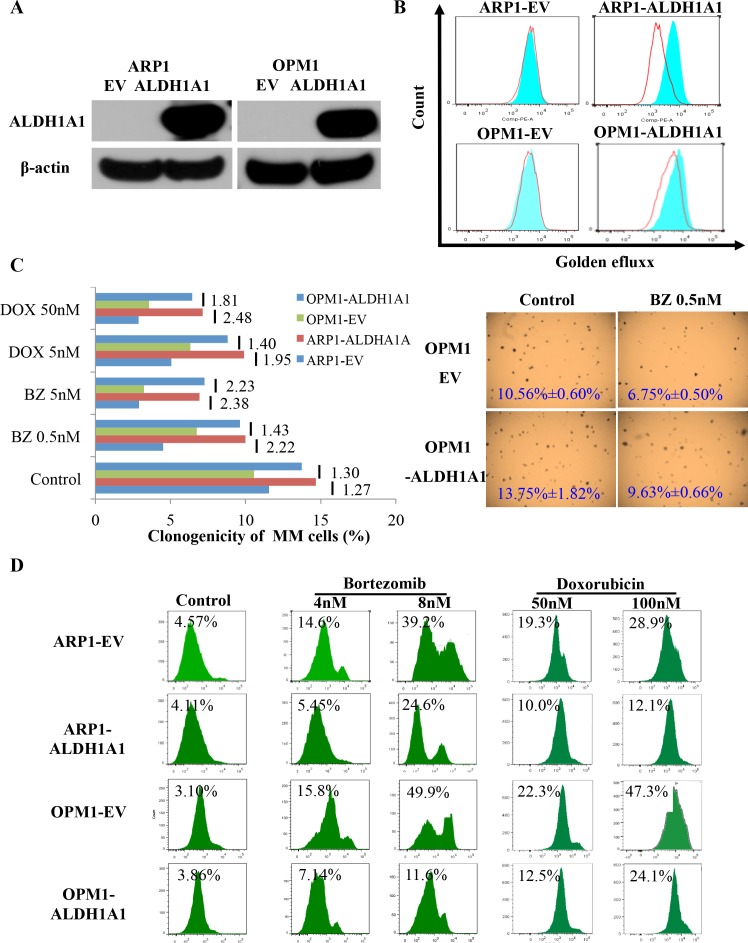
Enforced expression of ALDH1A1 in myeloma cells leads to myeloma drug resistance *in vitro* **(A)** Western blot demonstrating virus-dependent over-expression of ALDH1A1 protein in ARP1 and OPM1 myeloma cells. Cells were transfected with lentivirus that contained an ALDH1A1 expression cassette (OE) or did not (EV). **(B)** Flow-cytometric detection of increased drug-efflux capacity of ALDH1A1^OE^ myeloma cells compared to ALDH1A1^EV^ controls. Cells were treated with an inhibitor of the drug-efflux pump, ABCB1 (blue histograms), or left untreated (red histograms). The eFluxx-ID™ multidrug resistance assay was performed and the mean fluorescence intensity, MFI, a quantitative measure of multidrug resistance, was determined. The MFI increased by 50 and 32 fluorescence units in ARP1 and OPM1 cells, respectively. **(C)** Clonogenic assay showed that over-expression of ALDH1A1 promoted colony formation in ARP1 and OPM1 cell lines. Bar diagram (left) depicts percent clonogenic growth of ARP1 and OPM1 myeloma cells that either over-express ALDH1A1 (OE) or empty vector (EV). X axis presents the colony formation percentage, and Y axis shows different concentrations of drug treatment. Cells were treated using 0.5 nM or 5 nM bortezomib (Bz), 5 nM or 50 nM doxorubicin (Dox) after 1 week culture. The ratio of clonogenic expansion of paired ALDH1A1OE and ALDH1A1EV samples, subjected to the same treatment, is indicated beside short vertical lines; e.g., after exposure to 5 nM Bz, OPM1 ALDH1A1OE cells generated 2.23 times more colonies than ALDH1A1EV cells. Shown to the right are representative photomicroscopic images of two soft-agar dishes that contain myeloma cell colonies derived from untreated or Bz-treated OPM1 ALDH1A1OE and ALDH1A1EV cells. **(D)** Elevated tolerance of ALDH1A1^OE^ myeloma cells to myeloma drugs. ALDH1A1^OE^ ARP1 and OPM1 cells, and their ALDH1A1^EV^ counterparts (used as controls), were treated with indicated amounts of bortezomib (columns 2 and 3) or doxorubicin (columns 4 and 5) or left untreated (column 1). Percentage of dead cells (indicated above histograms) was determined by flow cytometry using APC-conjugated antibody to annexin V.

To assess whether enforced expression of ALDH1A1 in myeloma cells leads to heightened tolerance to myeloma drugs *in vitro*, we transduced ARP1 and OPM1 cells with a lentivirus-delivered, EF1-alpha promoter-driven full-length cDNA of ALDH1A1. As one would have expected, western analysis demonstrated over-expression of ALDH1A1 protein in both cell lines (designated ARP1^OE^ and OPM1^OE^) compared to controls transduced with non-coding “empty vector” (designated ARP1^EV^ and OPM1^EV^, Figure 2A). Next, we employed the eFluxx-ID™ multidrug resistance assay to evaluate the possibility that up-regulation of ALDH1A1 leads to increased drug efflux activity in myeloma. Cells were treated with specific inhibitors of 3 different drug export pumps; i.e., verapamil for ABCB1, MK-571 for ABCC1, and Novobiocin for ABCG2, or left untreated (control). Under conditions of ABCB1 (but not ABCC1 or ABCG2) inhibition, ARP1^OE^ and OPM1^OE^ cells exhibited significantly greater drug efflux activity than ARP1^EV^ and OPM1^EV^ cells: the mean fluorescence intensity (MFI) increased by 50 and 32 units, respectively (Figure 2B). The finding suggested that ALDH1A1 enhanced the drug export activity of myeloma cells in an ABCB1-dependent manner. Next we performed soft-agar colony formation assays to assess the possibility that ALDH1A1 promotes drug resistance in myeloma. Figure 2C shows that ARP1^OE^ and OPM1^OE^ cells were less sensitive to bortezomib (Bz) and doxorubicin (Dox) than ARP1^EV^ and OPM1^EV^ cells. Flow cytometric determination of immunoreactivity to annexin V, a marker of apoptotic cell death, revealed the same picture, as treatment of cells for 48 hrs with Bz (4 nM or 8 nM) or Dox (50 nM or 100 nM) caused less death in the “OE” than “EV” sample (Figure 2D). The results presented in panels C and D lent further support to the contention that ALDH1A1 renders myeloma cell drug resistance.

### Enforced expression of ALDH1A1 increases tolerance to myeloma drugs *in vivo*

**Figure 3:**
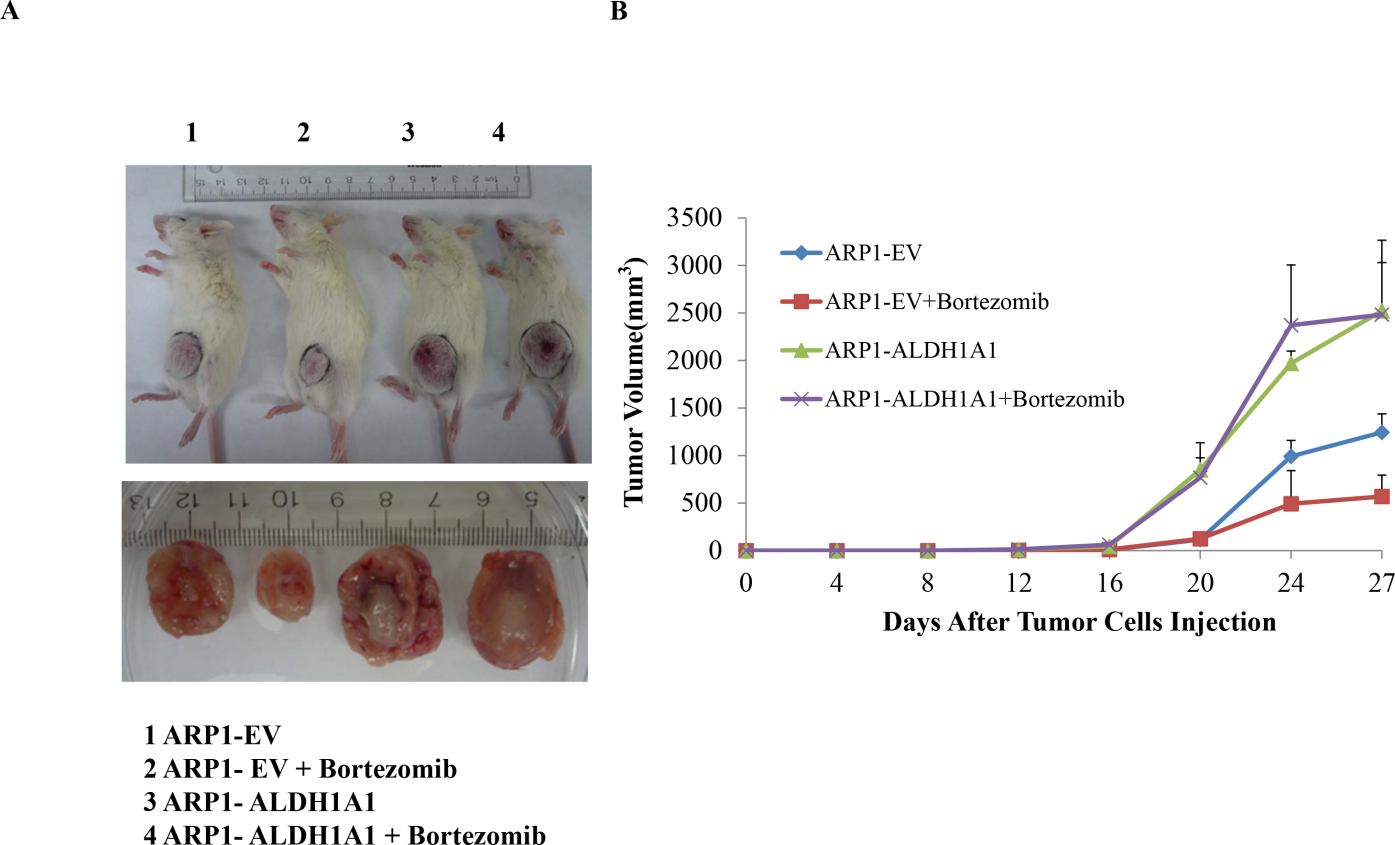
Over-expression of ALDH1A1 in myeloma cells induces myeloma drug resistance *in vivo* **(A)** Accelerated growth and reduced drug response of ALDH1A1^OE^ tumors relative to ALDH1A1^EV^ tumors. ARP1 cells over-expressed ALDH1A1 (OE) or transfected with empty vector (EV) were injected subcutaneously into NOD.Cγ-Rag1 mice, which were either treated with bortezomib or left untreated. All experimental groups contained 5 mice each. (p<0.05). **(B)** Increased size and smaller drug-induced inhibition of ALDH1A1^OE^ tumors relative to ALDH1A1^EV^ tumors. Tumors were harvested on day 27 after cell transfer injection and tumor volume was measured and calculated in the indicated groups from day 12 (started the treatment) to day 27 after injection

To extend these observations to an animal model of human myeloma, we transferred ARP1^OE^ and ARP1^EV^ cells to immunodeficient NOD.Cγ Rag1 mice. Starting on day 7 post transfer of 1.5 × 10^6^ tumor cells, half the mice were treated with Bz (1 mg/kg, twice weekly IP) and half were left untreated (control). Figure 3 shows that 27 days after cell propagation, the ARP1^OE^ tumors that developed in untreated mice were significantly larger (2.52 cm^3^ on average; n=5) than the ARP1^EV^ tumors (1.24 cm^3^). Similarly, in Bz-treated mice, ARP1^OE^ tumors were larger (2.48 cm^3^) than ARP1^EV^ tumors (0.57 cm^3^). We conclude that treatment with Bz had little if any impact on ARP1^OE^ tumors (1.6% difference in tumor volume in treated vs. untreated mice) but was highly effective in case of ARP1^EV^ tumors (54% difference). This result provided evidence that up-regulation of ALDH1A1 in myeloma mitigates the response to treatment with proteasome inhibitor.

### NEK2 is involved in the mechanism by which ALDH1A1 promotes drug resistance in myeloma

**Figure 4:**
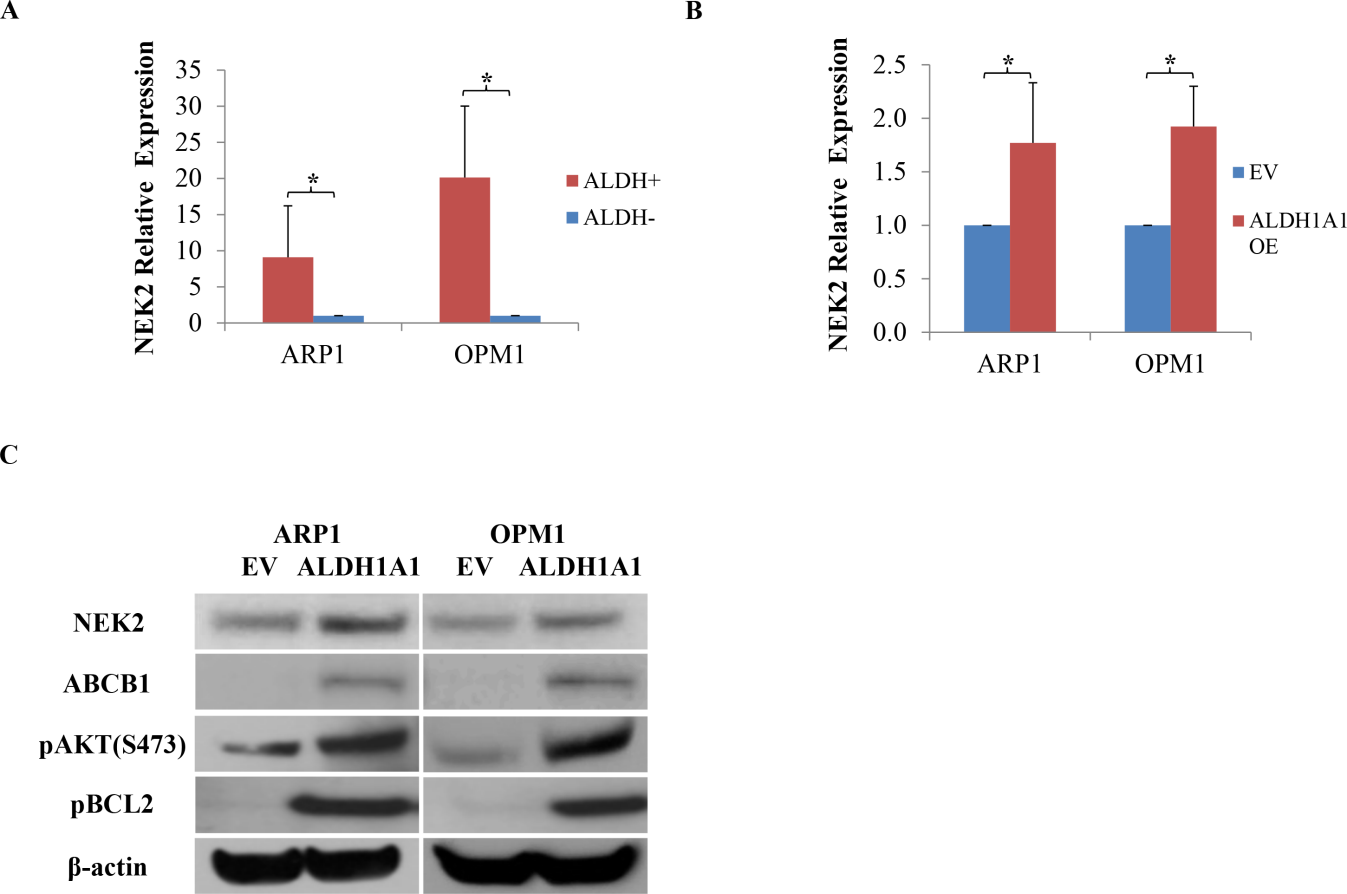
Enforced expression of ALDH1A1 in myeloma cells leads to up-regulation of genes implicated in myeloma drug resistance **(A)** *NEK2* expression, determined by qPCR, in ALDH1^+^ and ALDH1^−^ ARP1 and OPM1 myeloma cells. **(B)** *NEK2* expression, determined by qPCR, in ARP1 and OPM1 myeloma cells transfected with cDNA of ALDH1A1 (OE) or empty vector (EV). **(C)** Immunoblot analysis of proteins implicated in drug resistance in myeloma (NEK2, ABCB1, pAKT, pBCL2) or used as loading control (β-actin).

**Figure 5:**
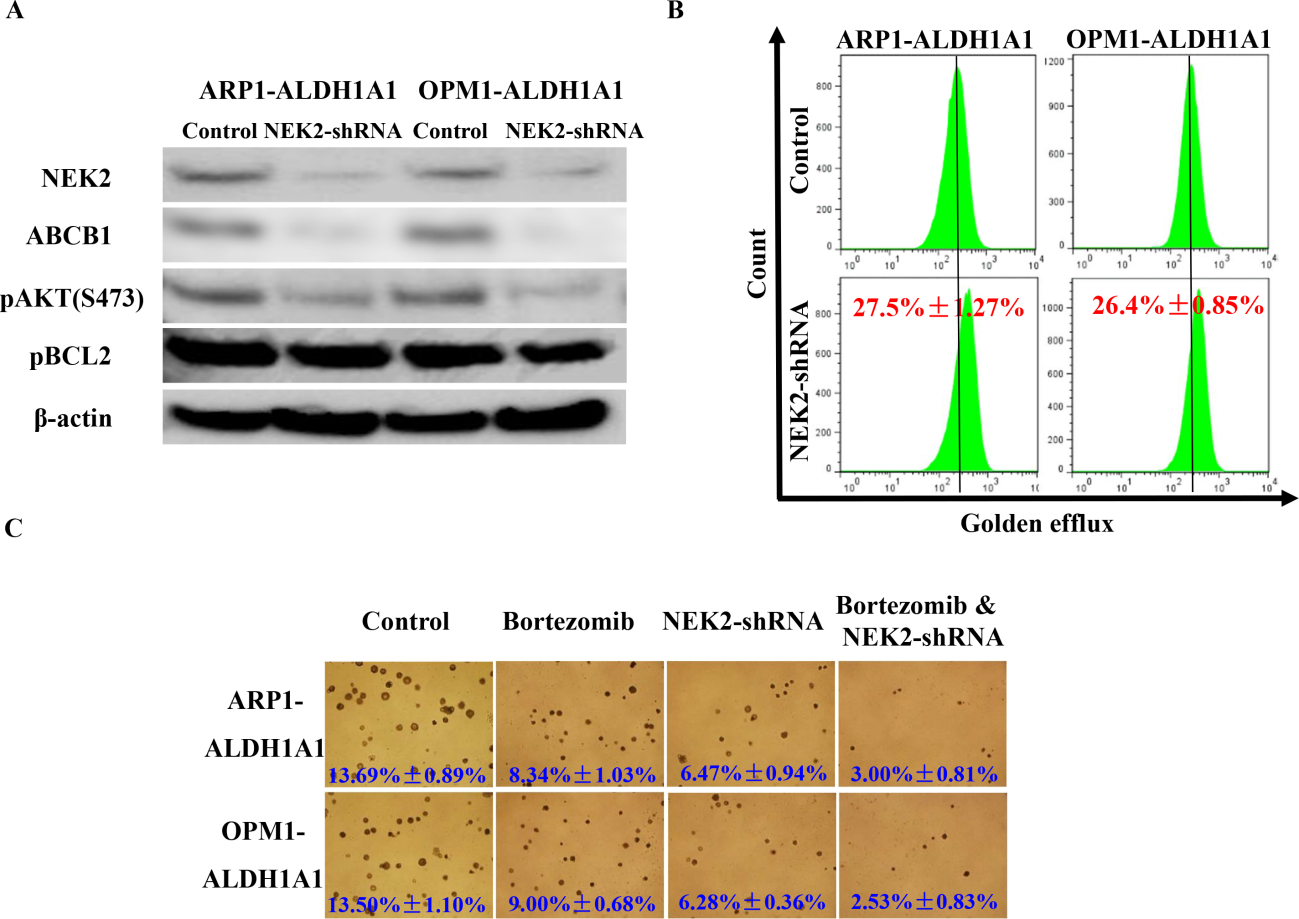
Knockdown of NEK2 expression myeloma cells that express high levels of ALDH1A1 leads to increased sensitivity to proteasome inhibition **(A)** Western blot demonstrating that doxycycline-inducible down-regulation of NEK2 by means of shRNA results in decreased ABCB1, pAKT and pBCL2 protein levels in myeloma. **(B)** Flow histograms indicating diminished ABCB1 drug efflux capacity in myeloma cells harboring reduced levels of NEK2. **(C)** Soft agar clonogenicity assays showing that “knock down” of NEK2 expression renders myeloma cells more sensitive to bortezomib than myeloma cells with unchanged NEK2 expression (original magnification of photographic images 40x).

Following up on our previous work demonstrating that up-regulation of NEK2 leads to therapy resistance and inferior survival in patients with MM [14], we decided to explore whether NEK2 might be involved in the mechanism by which ALDH1A1 promotes drug resistance in myeloma. NEK2 gene expression was found significantly higher in ALDH1^+^ than in ALDH1^−^ MM cells, fractionated by using Aldefluor®-based cell sorting from 3 MM lines [14]. qPCR analysis confirmed this finding (Figure 4A) and further demonstrated that high expression of *NEK2* is also a feature of ARP1^OE^ / OPM1^OE^ relative to ARP1^EV^ / OPM1^EV^ cells (Figure 4B). Because NEK2 promotes drug resistance by virtue of activating the myeloma drug efflux pump, ABCB1, in dependence on pAKT and pBCL2 levels [14], we decided to compare the level of these proteins in ARP1 and OPM2 cells that either over-expressed ALDH1A1 (OE) or transfected with empty vector (EV). Figure 4C shows that compared to EV cells, OE cells harbored higher levels of NEK2, ABCB1, pAKT and pBCL2 by western blotting. To further validate the involvement of NEK2 in ALDH1A1-induced drug resistance, *NEK2*-targeting shRNA was transduced into ALDH1A1-overexpressing ARP1 and OPM1 cells. In both cases, this led to reduced levels of NEK2, ABCB1, pAKT and pBCL2 proteins by western analysis (Figure 5A), decreased activity of the drug efflux pump by eFluxx-ID analysis (Figure 5B) and lessened colony formation upon treatment with bortezomib (5nM) or without treatment (Figure 5C). The apparent association of high ALDH1A1 and NEK2 levels is consistent with the hypothesis that NEK2 contributes to ALDH1A1-dependent drug resistance in myeloma.

### NEK2 is activated by 9-cis retinoic acid-dependent RXRα signaling in myeloma

**Figure 6:**
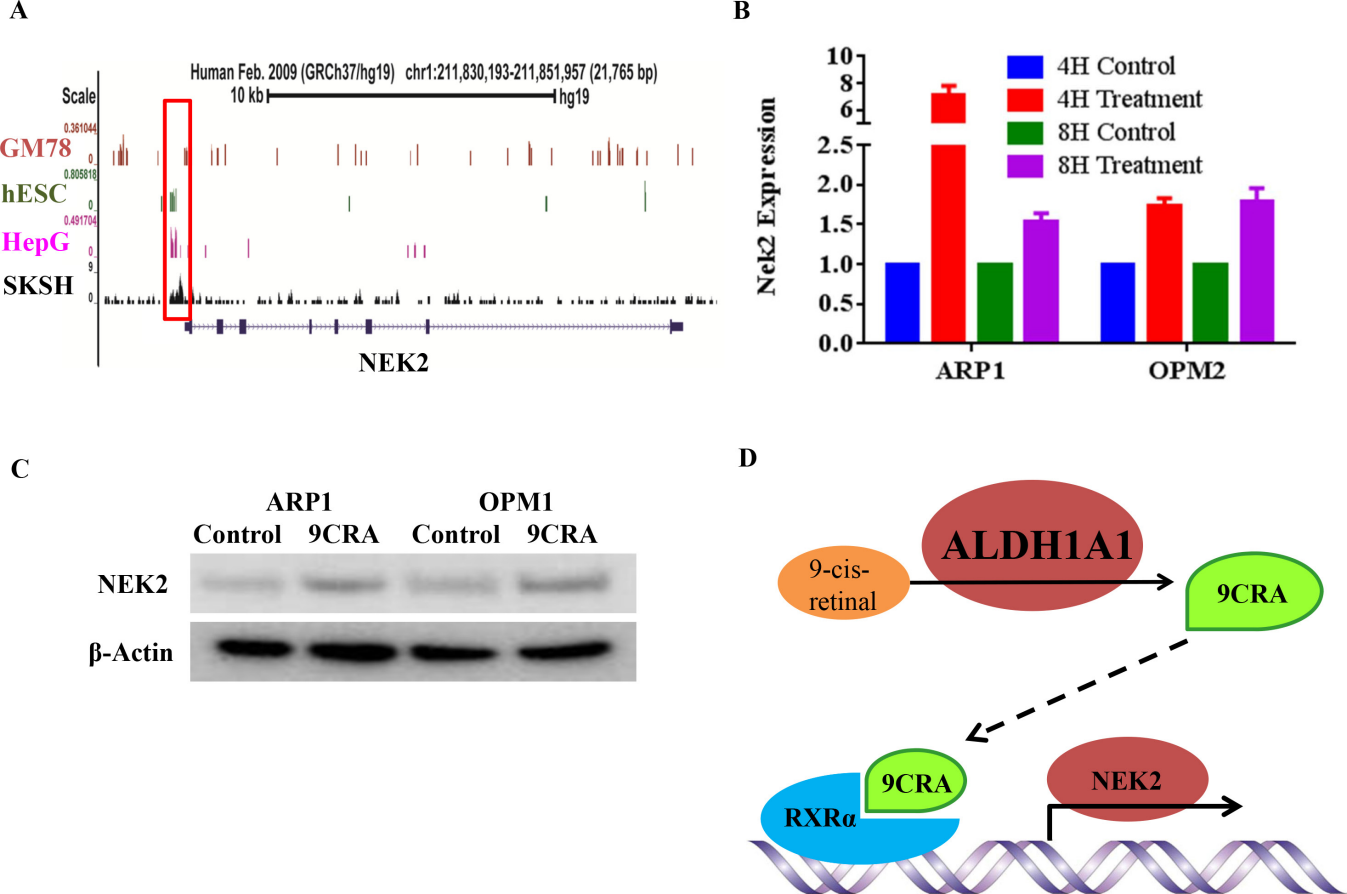
ALDH1A1-dependent regulation of NEK2 expression via retinoic acid signaling **(A)** RXRα chromatin occupancy pattern at the *NEK2* locus revealed by ChIP-Seq analysis of different four cell lines (indicated to the left of the black vertical line). The NEK2 promoter region is boxed. The NEK2 coding region is indicated by a thin, labeled horizontal line that contains small boxes, which denote exons. **(B)** qPCR results indicating increased NEK2 expression following treatment of cells with 9CRA (5nM) for 4 hours or 8 hours. **(C)** Western blot analysis of NEK2 protein levels in 2 myeloma cell lines treated with 9CRA (5nM) for 48 hrs or left untreated. **(D)** Working model on the putative mechanisms by which ALDH1A1 activates NEK2 and, thereby, promotes drug resistance in myeloma.

To elucidate the mechanism by which ALDH1A1 activates NEK2 in myeloma, we interrogated the chromatin immunoprecipitation sequencing (ChIP-Seq) database from the University of California Santa Cruz (UCSC) for chromatin occupancy patterns at the *NEK2* promoter and *NEK2* coding region. This revealed a significant footprint of retinoic X receptor alpha (RXRα) binding in 4 of 4 cell lines for which this information was available: GM78, hESC, HepG and SKSH (Figure 6A). Because RXRα is the receptor for 9-cis retinoic acid (9CRA), an important ligand of the cellular RXRα signal transduction pathway, the ChIP-Seq result suggested that ALDH1A1 regulates *NEK2* transcription by generating 9CRA. To follow up on this hypothesis, we treated APR1 and OPM1 cells for 4 or 8 hrs with 5nM 9CRA *in vitro*. qPCR analysis demonstrated heightened expression of *NEK2* in all cases, with a particularly strong increase in the 4-hr ARP1 sample (Figure 6B). Western blotting of whole cell lysates after treatment of cells with 5nM 9CRA for 48 hrs confirmed the PCR result at the protein level (Figure 6C). The data presented in Figure 6 lends credence to a model that implicates RXRα/9CRA-dependent transactivation of NEK2 in myeloma drug resistance RXRα (Figure 6D).

## DISCUSSION

MM is a difficult-to-treat malignancy of terminally differentiated B-lymphocytes that exhibits complex genetic/epigentic abnormalities and frequently evolves into treatment-refractory fatal disease [9]. Karyotypic changes are detected in ~30% of newly diagnosed patients, with number and complexity of changes correlating with disease stage, prognosis and response to therapy [32]. Comparing microarray-based gene expression profiles of myeloma samples serially collected from the same patients at different disease progression and treatment stages, we found the chromosomal instability (CIN) gene signature strongly increased after induction chemo- and tandem ASCT therapy, and tightly linked to poor prognosis of patients with myeloma [13]. More recently, we showed that ALDH1 is a marker of tumor initiating cells and drug resistance in myeloma, and that the CIN signature is more highly expressed in ALDH1^+^ than ALDH1^−^ myeloma cells [14]. Consistent with that, CD138^−^ALDH1^+^ cells were found to be more clonogenic and tumorigenic than CD138^+^ALDH1^−^ cells [33]. Because ALDH1 positivity, as determined by Aldefluor analysis, reflects the combined output of different aldehyde dehydrogenase activities – namely ALDH1A1, ALDH1B1 and ALDH1A3 et al. – we here dissected the ALDH1^+^ phenotype and demonstrated that ALDH1A1 is the predominant isoform in myeloma. Additionally, we showed that enforced expression of ALDH1A1 in two myeloma cell lines (ARP1, OPM1) led to both increased clono- and tumorigenicity and heightened tolerance to two widely used myeloma drugs (bortezomib, doxorubicin).

Our previous study demonstrated that as many as 5 of 17 genes found to be significantly up-regulated in ALDH1^+^ myeloma cells encode cell cycle-dependent protein kinases; specifically, CDC2, TTK, AURKA, AURKB and, of importance here, NEK2 [14]. Because cell cycle-dependent protein kinases are thought to be a major underlying reason for CIN and drug resistance in cancer [13, 14, 34], we hypothesized that NEK2 might be involved in the mechanism by which ALDH1A1 promotes therapy resistance in myeloma. This hypothesis was in line with evidence that up-regulation of NEK2 in myeloma prognosticates inferior survival and is strongly associated with CIN and drug resistance [13]. We found that ALDH1A1 induced NEK2 expression at the mRNA and protein levels. Furthermore, shRNA-dependent “knockdown” of NEK2 led to a drop of proteins crucial for cellular drug export (ABCB1) and survival (pAKT, pBCL2). Considering that NEK2 activates AKT and ABCB1 in myeloma [13], it is possible that ALDH1A1 promotes drug resistance by activating the NEK2-AKT pathway. It is also possible that the activation (phosphorylation) of BCL2 in ALDH1^+^ myeloma cells is a consequence of heightened CIN-NEK2-AKT signaling. BCL2 is a pro-survival protein that protects myeloma cells from drug-induced death [35] and is often over-expressed in cancer cells exhibiting the CIN phenotype, even though it should be down regulated following drug-dependent mitotic arrest [15, 36, 37]. Additional studies are warranted to elucidate the regulation of BCL2 in response to CIN-NEK2-AKT activation.

ALDH1A1 catalyzes the oxidation of retinal (retinaldehyde) to the corresponding retinoic acid (RA) [38], which – upon binding to retinoic acid receptors (RAR) and/or retinoid X receptors (RXR) – initiates downstream RA signaling. ALDH1A1-dependent generation of RA is important for growth and differentiation of normal and malignant cells [38, 39], including normal hematopoietic stem cells [38]. In embryonic cancer/stem cells and induced pluripotent stem (iPS) cells, RA signaling has been linked to CIN, based on increased occurrence of micronuclei and decreased expression of survivin [40]. Since ALDH1A1 generates two different RAs – the widely known all-trans retinoic acid (ATRA) and the lesser known 9-cis retinoic acid (9CRA) – and our previous work on myeloma pointed to ATRA as an important signaling ligand [39], this research concentrated initially on ATRA. However, treatment of myeloma cells with ATRA left NEK2 levels unchanged upon qPCR analysis (data not shown). We also investigated whether enforced up-regulation of the ATRA receptor, RARα, might make a difference – because RARα2 (not RARα1) is important for myeloma [39, 41], we used RARα2 for that purpose. We found no change in NEK2 using qPCR and western analyses (results not shown). These findings indicated that ATRA is not involved in the mechanism by which ALDH1 regulates NEK2 and led us to refocus attention on 9CRA. This resulted in the discovery that ALDH1A1 promotes *NEK2* transcription in a 9CRA/RXRα-dependent fashion. The result is in agreement with ChIP-Seq data gathered by other investigators, demonstrating NEK2 promoter occupancy by the 9CRA-specific receptor, RXRα, in different four cell lines.

In conclusion, this study implicates ALDH1A1 as an important drug resistance and tumor progression gene in multiple myeloma. The mechanism of ALDH1A1 includes up-regulation and activation of NEK2, AKT and BCL2, but many details remain outstanding. Small-drug inhibitors that specifically target ALDH1A1 may be considered for therapeutic regimens aimed at overcoming drug resistance in myeloma.

## MATERIALS AND METHODS

### Cell culture

Human myeloma cell lines, APR1 and OPM1 were cultured at 37°C and 5% CO_2_ in RPMI 1640 (Gibco, Grand Island, NY) supplemented with 10% heat-inactivated fetal calf serum (Gibco, Grand Island, NY) and penicillin and streptomycin (100 μg/mL, Sigma, St. Louis, MO).

### Reagents

Antibodies to NEK2 (sc-55601) and ABCB1 (sc-55510) were purchased from Santa Cruz Biotechnology (Dallas, Texas), and antibodies to ALDH1A1 (catalog number 12035), pAKT (3787), pBCL2 (2827) and β-actin (4967) from Cell Signaling Technology (Danvers, MA). All-trans retinoic acid, doxorubicin, doxycycline hyclate and 9-cis retinoic acid were procured from Sigma (St. Louis, MO). Bortezomib was obtained from Millennium (Cambridge, MA).

### ALDEFLUOR assay, flow cytometry, and fluorescence-activated cell sorting (FACS)

The ALDEFLUOR assay (Stem Cell Technologies, Vancouver, Canada) was performed according to the recommendations of the manufacturer. Briefly, one million cells were re-suspended in 1mL assay buffer and incubated with activated ALDEFLUOR substrate (5 μL) at 37°C in a water bath (30 min). Cells treated with diethylaminobenzaldehyde (DEAB, 5 μL) were used as control. Cells were centrifuged, re-suspended in ice-cold assay buffer, and either analyzed by flow cytometry or sorted using a FACS Aria (Becton Dickinson).

### Quantitative reverse-transcription PCR (qPCR)

Total RNA was extracted with the assistance of a RNeasy RNA isolation kit (Qiagen) and reverse transcribed using the SuperScript III RT kit and oligo dT primers (Invitrogen, Carlsbad, CA). PCR primers were purchased from Integrated DNA Technologies (Coralville, IA). Fold changes were calculated using the ΔΔC_t_ method and β-actin message as reference.

### Soft-agar clonogenicity assay

Clonogenic growth was evaluated in 12-well plates after seeding 10,000 myeloma cells in 0.5 mL 0.33% agar/RPMI 1640 (Invitrogen, Carlsbad, CA) supplemented with 10% FCS. Cells were incubated (37°C, 5% CO_2_) and fed with the same medium twice in the first week, and then treated with drugs or left untreated (control) for another 2 weeks. One colony was defined if more than 40 cells were observed. Plates were imaged and colonies were enumerated using Image J freeware. The latter entailed threshold adjustment using the Adjust Manu bar followed by colony counting using the Measure Manu bar [13].

### Western blots

Western blots were performed to determine expression levels of proteins in MM cells [39]. Briefly, cells were lysed using the Mammalian Cell Extraction Kit (K269–500) from Biovision (Milpitas, CA). 10 μg of protein were loaded, fractionated by SDS-PAGE in 4–12% polyacrylamide gels, and transferred to nitrocellulose. Membranes were blocked with 5% non-fat dry milk in Tris buffered saline (TBS) containing 0.05% Tween-20 (TBST) prior to incubation overnight at 4°C with primary antibodies. Protein bands were visualized using HRP-conjugated secondary antibodies and SuperSignal West Pico (Pierce, Rockford, IL). Blots were subsequently stripped and re-probed for β-actin as loading control.

### Gene expression profiling (GEP) and data analysis

GEP was performed on Affymetrix U133 Plus2.0 microarrays as previously described [13, 42]. GEP access number of primary myeloma sequential samples reported in this paper is GSE19554.

### Lentiviral gene transduction

Lentivirus containing cDNA gene expression or shRNA knockdown cassettes was constructed as described previously [43, 44]. The plasmid containing the human ALDH1A1 full-length open-reading frame, NM_000689, was provided by Health Sciences Center (HSC) Core Research Facilities, University of Utah. Primer sequences for PCR-based cloning of ALDH1A1 cDNA were as follows: 5′-GGG GTT TAA ACA TGT CAT CCT CAG GCA CGC CAG AG-3′ (forward) and 5′- GGG GTT TAA ACT TAT GAG TTC TTC TGA GAG ATT TTC ACT GTG AC-3′ (reverse). The ALDH1A1 coding sequence was cloned into the lentiviral vector, pWPI. NEK2-targeted shRNA was cloned into vector, pLVTH, using the following PCR primers: 5′-GAT CCC CGG AGG AAG AGT GAT GGC AAG ATT CAA GAG ATC TTG CCA TCA CTC TTC CTC CTT TTT A-3′ and a nonsense scrambled oligonucleotide (5′-GAT CCC CGA CAC GCG ACT TGT ACC ACT TCA AGA GAG TGG TAC AAG TCG CGT GTC TTT TTA-3′) were obtained from Integrated DNA Technologies (Coralville, IA). Recombinant lentivirus was produced in 293T cells. Transduction efficiency, determined by flow cytometry of fluorescent reporter gene, was ~95%.

### Apoptosis and dye-efflux multidrug-resistance assays

Programmed cell death was determined with the help of the flow-cytometric Annexin V apoptosis detection kit APC (catalog number 88–8007) from eBioscience (San Diego, CA). One million cells were washed in PBS and suspended in 1 mL binding buffer. Fluorochrome-conjugated antibody to Annexin V (5 μL) was added to the cell suspension (100 μL). Cells were incubated for 15 min at room temperature, washed and re-suspended in binding buffer and subjected to flow analysis. Drug resistance was measured using the eFluxx-ID™ Multidrug resistance assay as previously described [13]. Briefly, 500,000 cells were incubated (30 min, 37°C, water bath) with detection reagent, Golden dye, either with specific inhibitors of ABCB1, ABCC1 and ABCG2 drug export pumps or without inhibitors (control). Cells were washed and re-suspended in cold PBS for flow analysis, using MCF7 cells as reference.

### Human myeloma xenografts in mice

All animal work was performed in accordance with the guidelines of the Institutional Animal Care and Use Committee of the University of Iowa under Animal Study Protocol 1202033. ARP1-ALDH1A1^OE^ cells and ARP1^EV^ cells (1.5 × 10^6^) were injected subcutaneously in the abdominal area of 6–8-week old NOD.Cγ-Rag1 mice (Jackson laboratory, Bar Harbor, Maine). Beginning on day 7 post cell transfer, mice were treated with bortezomib (1 mg/kg IP) twice weekly until humane endpoints were reached. Tumor volume was measured using a caliper and mice were sacrificed by CO_2_ asphyxiation when tumor diameter reached 20 mm.

### Statistical analysis

All values were analyzed using two-tailed Student's t-test and expressed as mean ± SD. A *p* value of 5% (*, P < 0.05) was considered significant.

## References

[1] Anderson KC, Carrasco RD (2011). Pathogenesis of myeloma. Annual review of pathology.

[2] Dewald GW, Kyle RA, Hicks GA, Greipp PR (1985). The clinical significance of cytogenetic studies in 100 patients with multiple myeloma, plasma cell leukemia, or amyloidosis. Blood.

[3] Mohamed AN, Bentley G, Bonnett ML, Zonder J, Al-Katib A (2007). Chromosome aberrations in a series of 120 multiple myeloma cases with abnormal karyotypes. American journal of hematology.

[4] Nilsson T, Hoglund M, Lenhoff S, Rylander L, Turesson I, Westin J, Mitelman F, Johansson B (2003). A pooled analysis of karyotypic patterns, breakpoints and imbalances in 783 cytogenetically abnormal multiple myelomas reveals frequently involved chromosome segments as well as significant age- and sex-related differences. British journal of haematology.

[5] Sawyer JR, Waldron JA, Jagannath S, Barlogie B (1995). Cytogenetic findings in 200 patients with multiple myeloma. Cancer genetics and cytogenetics.

[6] Greenman C, Stephens P, Smith R, Dalgliesh GL, Hunter C, Bignell G, Davies H, Teague J, Butler A, Stevens C, Edkins S, O'Meara S, Vastrik I, Schmidt EE, Avis T, Barthorpe S (2007). Patterns of somatic mutation in human cancer genomes. Nature.

[7] Abdi J, Chen G, Chang H (2013). Drug resistance in multiple myeloma: latest findings and new concepts on molecular mechanisms. Oncotarget.

[8] Matsui W, Huff CA, Wang Q, Malehorn MT, Barber J, Tanhehco Y, Smith BD, Civin CI, Jones RJ (2004). Characterization of clonogenic multiple myeloma cells. Blood.

[9] Leung-Hagesteijn C, Erdmann N, Cheung G, Keats JJ, Stewart AK, Reece DE, Chung KC, Tiedemann RE (2013). Xbp1s-negative tumor B cells and pre-plasmablasts mediate therapeutic proteasome inhibitor resistance in multiple myeloma. Cancer cell.

[10] Matsui W, Wang Q, Barber JP, Brennan S, Smith BD, Borrello I, McNiece I, Lin L, Ambinder RF, Peacock C, Watkins DN, Huff CA, Jones RJ (2008). Clonogenic multiple myeloma progenitors, stem cell properties, and drug resistance. Cancer research.

[11] Siegel R, Naishadham D, Jemal A (2012). Cancer statistics, 2012. CA: a cancer journal for clinicians.

[12] Holohan C, Van Schaeybroeck S, Longley DB, Johnston PG (2013). Cancer drug resistance: an evolving paradigm. Nature reviews Cancer.

[13] Zhou W, Yang Y, Xia J, Wang H, Salama ME, Xiong W, Xu H, Shetty S, Chen T, Zeng Z (2013). NEK2 Induces Drug Resistance Mainly through Activation of Efflux Drug Pumps and Is Associated with Poor Prognosis in Myeloma and Other Cancers. Cancer cell.

[14] Zhou W, Yang Y, Gu Z, Wang H, Xia J, Wu X, Zhan X, Levasseur D, Zhou Y, Janz S, Tricot G, Shi J, Zhan F (2013). ALDH1 activity identifies tumor-initiating cells and links to chromosomal instability signatures in multiple myeloma. Leukemia.

[15] Lee AJ, Endesfelder D, Rowan AJ, Walther A, Birkbak NJ, Futreal PA, Downward J, Szallasi Z, Tomlinson IP, Howell M, Kschischo M, Swanton C (2011). Chromosomal instability confers intrinsic multidrug resistance. Cancer research.

[16] Carter SL, Eklund AC, Kohane IS, Harris LN, Szallasi Z (2006). A signature of chromosomal instability inferred from gene expression profiles predicts clinical outcome in multiple human cancers. Nature genetics.

[17] Koppaka V, Thompson DC, Chen Y, Ellermann M, Nicolaou KC, Juvonen RO, Petersen D, Deitrich RA, Hurley TD, Vasiliou V (2012). Aldehyde dehydrogenase inhibitors: a comprehensive review of the pharmacology, mechanism of action, substrate specificity, and clinical application. Pharmacological reviews.

[18] Druesne-Pecollo N, Tehard B, Mallet Y, Gerber M, Norat T, Hercberg S, Latino-Martel P (2009). Alcohol and genetic polymorphisms: effect on risk of alcohol-related cancer. The lancet oncology.

[19] Yu HS, Oyama T, Isse T, Kitakawa K, Ogawa M, Pham TT, Kawamoto T (2009). Characteristics of aldehyde dehydrogenase 2 (Aldh2) knockout mice. Toxicology mechanisms and methods.

[20] Langevin F, Crossan GP, Rosado IV, Arends MJ, Patel KJ (2011). Fancd2 counteracts the toxic effects of naturally produced aldehydes in mice. Nature.

[21] Rizzo WB (2007). Sjogren-Larsson syndrome: molecular genetics and biochemical pathogenesis of fatty aldehyde dehydrogenase deficiency. Molecular genetics and metabolism.

[22] Puttmann L, Stehr H, Garshasbi M, Hu H, Kahrizi K, Lipkowitz B, Jamali P, Tzschach A, Najmabadi H, Ropers HH, Musante L, Kuss AW (2013). A novel ALDH5A1 mutation is associated with succinic semialdehyde dehydrogenase deficiency and severe intellectual disability in an Iranian family. American journal of medical genetics Part A.

[23] Muzio G, Maggiora M, Paiuzzi E, Oraldi M, Canuto RA (2012). Aldehyde dehydrogenases and cell proliferation. Free radical biology & medicine.

[24] Bicknell LS, Pitt J, Aftimos S, Ramadas R, Maw MA, Robertson SP (2008). A missense mutation in ALDH18A1, encoding Delta1-pyrroline-5-carboxylate synthase (P5CS), causes an autosomal recessive neurocutaneous syndrome. European journal of human genetics : EJHG.

[25] Ho KK, Mukhopadhyay A, Li YF, Mukhopadhyay S, Weiner H (2008). A point mutation produced a class 3 aldehyde dehydrogenase with increased protective ability against the killing effect of cyclophosphamide. Biochemical pharmacology.

[26] Moreb JS, Ucar D, Han S, Amory JK, Goldstein AS, Ostmark B, Chang LJ (2012). The enzymatic activity of human aldehyde dehydrogenases 1A2 and 2 (ALDH1A2 and ALDH2) is detected by Aldefluor, inhibited by diethylaminobenzaldehyde and has significant effects on cell proliferation and drug resistance. Chemico-biological interactions.

[27] Januchowski R, Wojtowicz K, Zabel M (2013). The role of aldehyde dehydrogenase (ALDH) in cancer drug resistance. Biomedicine & pharmacotherapy = Biomedecine & pharmacotherapie.

[28] Sullivan JP, Spinola M, Dodge M, Raso MG, Behrens C, Gao B, Schuster K, Shao C, Larsen JE, Sullivan LA, Honorio S, Xie Y, Scaglioni PP, DiMaio JM, Gazdar AF, Shay JW (2010). Aldehyde dehydrogenase activity selects for lung adenocarcinoma stem cells dependent on notch signaling. Cancer research.

[29] Luo Y, Dallaglio K, Chen Y, Robinson WA, Robinson SE, McCarter MD, Wang J, Gonzalez R, Thompson DC, Norris DA, Roop DR, Vasiliou V, Fujita M (2012). ALDH1A isozymes are markers of human melanoma stem cells and potential therapeutic targets. Stem cells (Dayton, Ohio).

[30] Charafe-Jauffret E, Ginestier C, Iovino F, Tarpin C, Diebel M, Esterni B, Houvenaeghel G, Extra JM, Bertucci F, Jacquemier J, Xerri L, Dontu G, Stassi G, Xiao Y, Barsky SH, Birnbaum D (2010). Aldehyde dehydrogenase 1- positive cancer stem cells mediate metastasis and poor clinical outcome in inflammatory breast cancer. Clinical cancer research : an official journal of the American Association for Cancer Research.

[31] Choi I, Lee S, Kyoung Chung H, Suk Lee Y, Eui Kim K, Choi D, Park EK, Yang D, Ecoiffier T, Monahan J, Chen W, Aguilar B, Lee HN, Yoo J, Koh CJ, Chen L (2012). 9-cis retinoic acid promotes lymphangiogenesis and enhances lymphatic vessel regeneration: therapeutic implications of 9-cis retinoic acid for secondary lymphedema. Circulation.

[32] Mahindra A, Hideshima T, Anderson KC (2010). Multiple myeloma: biology of the disease. Blood reviews.

[33] Reghunathan R, Bi C, Liu SC, Loong KT, Chung TH, Huang G, Chng WJ (2013). Clonogenic multiple myeloma cells have shared stemness signature assocuated with patient survival. Oncotarget.

[34] Hayward DG, Fry AM (2006). Nek2 kinase in chromosome instability and cancer. Cancer letters.

[35] Chanan-Khan A (2005). Bcl-2 antisense therapy in B-cell malignancies. Blood reviews.

[36] Blagosklonny MV (2007). Mitotic arrest and cell fate: why and how mitotic inhibition of transcription drives mutually exclusive events. Cell cycle (Georgetown, Tex).

[37] Swanton C, Nicke B, Schuett M, Eklund AC, Ng C, Li Q, Hardcastle T, Lee A, Roy R, East P, Kschischo M, Endesfelder D, Wylie P, Kim SN, Chen JG, Howell M (2009). Chromosomal instability determines taxane response. Proceedings of the National Academy of Sciences of the United States of America.

[38] Allahverdiyev AM, Bagirova M, Oztel ON, Yaman S, Abamor ES, Koc RC, Ates SC, Elcicek S, Baydar SY (2012). Aldehyde Dehydrogenase: Cancer and Stem Cells.

[39] Yang Y, Shi J, Tolomelli G, Xu H, Xia J, Wang H, Zhou W, Zhou Y, Das S, Gu Z (2013). RARα2 expression confers myeloma stem cell features. Blood.

[40] Sartore RC, Campos PB, Trujillo CA, Ramalho BL, Negraes PD, Paulsen BS, Meletti T, Costa ES, Chicaybam L, Bonamino MH, Ulrich H, Rehen SK (2011). Retinoic acid-treated pluripotent stem cells undergoing neurogenesis present increased aneuploidy and micronuclei formation. PloS one.

[41] Wang S, Tricot G, Shi L, Xiong W, Zeng Z, Xu H, Zangari M, Barlogie B, Shaughnessy JD, Zhan F (2009). RARalpha2 expression is associated with disease progression and plays a crucial role in efficacy of ATRA treatment in myeloma. Blood.

[42] Zhan F, Huang Y, Colla S, Stewart JP, Hanamura I, Gupta S, Epstein J, Yaccoby S, Sawyer J, Burington B, Anaissie E, Hollmig K, Pineda-Roman M, Tricot G, van Rhee F, Walker R (2006). The molecular classification of multiple myeloma. Blood.

[43] Shi L, Wang S, Zangari M, Xu H, Cao TM, Xu C, Wu Y, Xiao F, Liu Y, Yang Y (2010). Over-expression of CKS1B activates both MEK/ERK and JAK/STAT3 signaling pathways and promotes myeloma cell drug-resistance. Oncotarget.

[44] Zhan F, Colla S, Wu X, Chen B, Stewart JP, Kuehl WM, Barlogie B, Shaughnessy JD (2007). CKS1B, overexpressed in aggressive disease, regulates multiple myeloma growth and survival through SKP2- and p27Kip1- dependent and - independent mechanisms. Blood.

